# Differential gene expression and clonal selection during cellular transformation induced by adhesion deprivation

**DOI:** 10.1186/1471-2121-11-93

**Published:** 2010-12-02

**Authors:** Rajeswari Jinka, Renu Kapoor, Sivapriya Pavuluri, Avinash T Raj, Mahesh J Kumar, Lakshmi Rao, Gopal Pande

**Affiliations:** 1 Acharya Nagarjuna University, Department of Biochemistry, Guntur - 522510, India; 2 Centre for Cellular and Molecular Biology, Uppal Road, Hyderabad - 500 007, India; 3 Vimta Labs Limited, Life Sciences Campus, #5, Alexandria Knowledge Park, Genome Valley, Shameerpet, Hyderabad - 500 078, India

## Abstract

**Background:**

Anchorage independent growth is an important hallmark of oncogenic transformation. Previous studies have shown that when adhesion dependent fibroblasts were prevented from adhering to a substrate they underwent anoikis. In the present study we have demonstrated how anoikis resistant cells gain the transformation related properties with sequential selection of genes. We have proposed this process as a model system for selection of transformed cells from normal cells.

**Results:**

This report demonstrates that some fibroblasts can survive during late stages of anoikis, at which time they exhibit transformation-associated properties such as *in vitro *colony formation in soft agar and *in vivo *subcutaneous tumour formation in nude mice. Cytogenetic characterisation of these cells revealed that they contained a t (2; 2) derivative chromosome and they have a selective survival advantage in non adherent conditions. Gene expression profile indicated that these cells over expressed genes related to hypoxia, glycolysis and tumor suppression/metastasis which could be helpful in their retaining a transformed phenotype.

**Conclusion:**

Our results reveal some new links between anoikis and cell transformation and they provide a reproducible model system which can potentially be useful to study multistage cancer and to identify new targets for drug development.

## Background

The process of cellular transformation involves the conversion of normal cells into malignant cells by acquisition of distinctive features such as indefinite life span, anchorage and serum independent growth, tumor formation, chromosomal abnormalities and altered gene expression [1]. It has been shown that if adhesion dependent normal cells are not allowed to adhere to the substratum, they undergo anoikis; however, many cancer cells can survive without adhering strongly to a substratum [2,3]. Several other studies have also shown that signals related to cell-cell and cell-substrate adhesion play a role in cellular transformation [4]. Oba-Shinjo et al., [5] have shown that sequential cycles of forced anchorage inhibition can act as a stimulus for development of cancer. Thus, anchorage independent growth plays a pivotal role in developing cancer cell invasion and metastasis and it is considered as a hallmark of cellular transformation [6].

In addition to the above, many genetic alterations also increase the proliferative potential of normal cells and desensitize them to signals that normally inhibit growth or promote cell death. These changes, which initially lead to the formation of tumors, can later also, be responsible for their metastatic spread [7]. More recently, it has been shown that initiation of metastatic events in tumors depends on certain specialized cancer cells that are referred to as cancer stem cells. These cells are capable of multipotent cell differentiation which implies that they can self renew and they can produce more malignant cells that migrate to other tissues [8,9]. It has been reported that the tumor phenotype and its cellular heterogeneity are modulated by the microenvironment, which comprises stromal interactions, hypoxia, paucity of nutrients, impaired differentiation, and activation of epithelial-mesenchymal transition (EMT) associated pathways [10]. Chromosomal non-disjunction and the subsequent production of aneuploid daughter cells during mitosis is also a common characteristic of tumour progression [11].

The availability of xenograft tumor models has enabled the verification of the above-mentioned observations and it has allowed us to study the *in vivo *facets of tumorigenesis, which include angiogenesis and metastasis. Although several xenograft models are available, the genetic and epigenetic alterations associated with the conversion of tumorigenic to metastatic cells are only poorly understood [12].

We have developed a xenograft tumour model with transformed rat fibroblasts, which are obtained by rescuing cells from the process of anoikis. This model is based upon our earlier studies on the signalling properties of these cells during anoikis [13,14]. In our model we have demonstrated that cells that can survive the process of anoikis in the early stages exhibit properties of transformed cells such as tumour formation and chromosomal instability. They also express genes correlated with the metastatic properties of cancer cells. We propose that this model could be very useful in understanding the relationship between anoikis and cell transformation and it could be used to identify new targets for drug development to prevent cancer metastasis.

## Results

### Cell viability and Transformation

**Figure 1:**
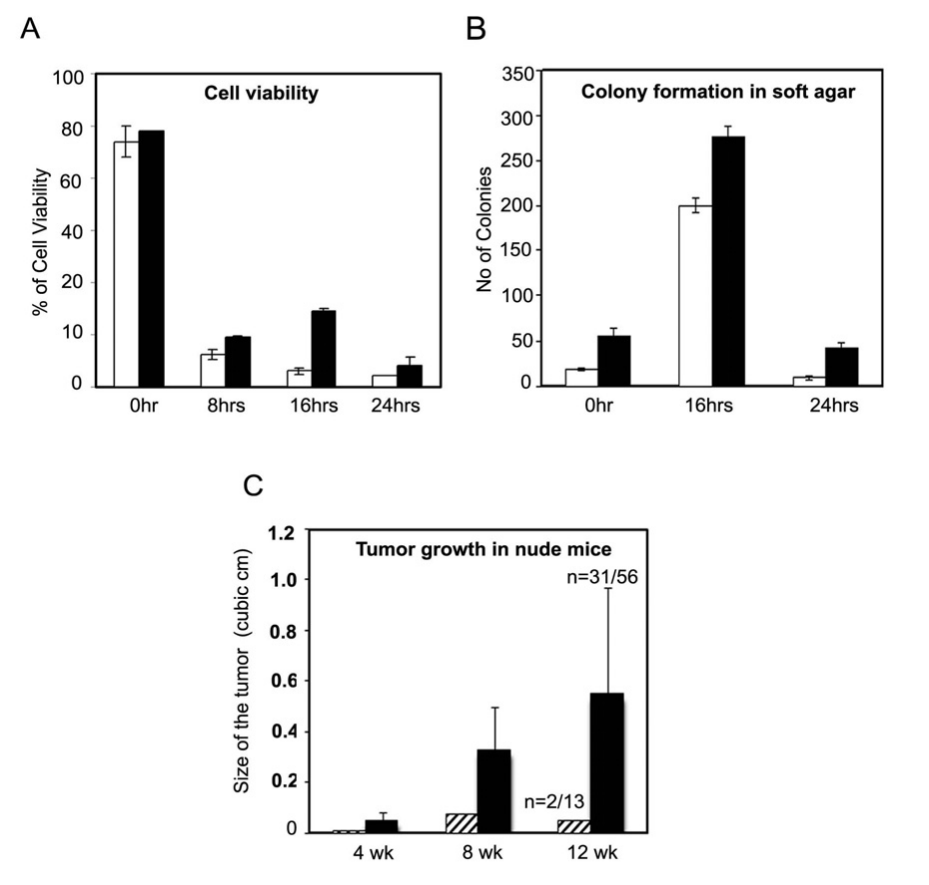
**Cell viability and transformation assays**. A. **Cell viability of Primary and F111 cells**. Cells were collected by trypsinization and plated on agar-coated surfaces at their represented time periods and assayed for viability by using MTT. Loss of cell viability is progressed with the time as shown in the figure with unfilled and filled bars representing Primary and F111 cells. B. **Soft agar colony formation of primary and F111 cells. **1×10^5 ^cells collected at the indicated time periods were mixed with 0.3% of agar and overlaid on 1% agar. The colonies with more than 50 cells were accounted and the number was high at 16 h time period as indicated in the figure. Unfilled and filled bars in the figure represent Primary and F111 cells. C. **Tumour formation assay in nude mice**. Adherent for 16 hrs (A16) (crossed bars) and non-adherent for 16 hrs (NA16) (Filled bars) cells derived from F111 cells were injected subcutaneously in to nude mice and the size of the tumour increased with F111 NA16 cells was measured at indicated time periods and average size of the tumour is represented in the figure.

We estimated the viability of F111 fibroblast cell line (Additional File 1 Figure S1a) and skin fibroblast primary culture (PC) cells (Additional File 1 Figure S1b), under adherent and non-adherent conditions. When cells, from either cell type were incubated on agarose-coated surfaces, they lost their viability very significantly with passage of time, as shown in Figure 1A. Approximately, 15-20% F111 cells and 3-5% PC cells remained viable even after remaining suspended for 16 h; but an insignificant number of F111 NA24 cells (< 4%) were viable after 24 hrs. The surviving cells after remaining suspended for 16 h on agarose have been referred in text as F111 NA16 (Additional File 1 Figure S1c) cells and PC NA16 (Additional File 1 Figure S1d) cells respectively.

F111 NA16 and PC NA16 cells were subjected to the soft agar colony formation assay as described and the results are shown in Figure 1B. The colonies obtained from both cell types are shown in Additional File 1 Figure S1e&f. We observe only background of 20 colonies from adherent PC cells (0 h), but with PC NA16 cells approximately 200 colonies/1×10^5 ^viable cells were observed. F111 A16 cells showed 56 colonies, which increased to 274 colonies in F111 NA16 cells. With NA 24 cells, derived either from PC or F111 cells, a very small number (< 30) of colonies were seen due to loss of viability to near zero levels. These results clearly show that both PC and F111 cells exhibit the transformed cell phenotype in their NA16 but not in the A16 counterpart. The colonies seen in F111 A16 cultures could be due to some partially transformed cells, containing chromosomal changes, which were present in the original cultures (see Discussion).

### Tumour formation in nude mice

To further confirm the transformed status of PC NA16 and F111 NA16 cells, all cell types were injected subcutaneously in to 6-8 week old homozygous nude mice (Nu/Nu). Tumour formation was measured at weekly intervals. PC cells did not show any tumour growth with either A16 [3 animals] or NA16 cells [3 animals] (Figure 1C). With F111 A16 cells, we observed only a small nodule like outgrowth after 4-6 weeks post injection in 2 out of 13 animals. This nodule did not grow further and regressed. In F111 NA16 cells, 31 out of 56 animals showed tumour growth after 4 weeks post injection and tumors grew rapidly and reached to a size of approximately 0.55 ± 0.05 cm^3 ^within 12 weeks, after which tumor-bearing animals died.

Histopathological examination of tumours and other tissues in these 31 animals was done by H&E and collagen specific Masson-Trichome staining. In all tumor sections, we found that the injected cells grew densely as spindle shaped neoplastic cells with crescent shaped nuclei and high amount of collagen secretion, indicative of fibrosarcomatous growth (Additional File 2 Figure S2A, D). Staining pattern of lung and liver sections from these animals showed that these neoplastic cells with crescent shaped nuclei, invaded into the lung and liver between 8-12 weeks of post injection (Additional File 2 Figure S2B, C, E&F), indicating the metastatic nature of the tumour.

### Chromosomal abnormalities and clonal selection

**Figure 2:**
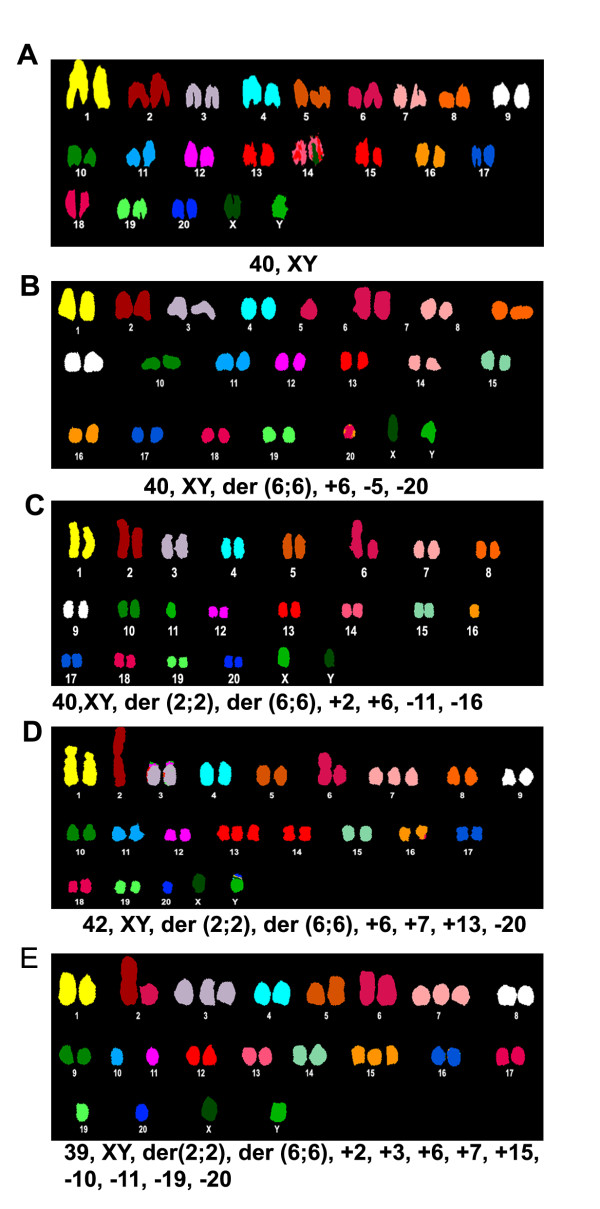
**Spectrally classified karyotypic images**. Spectrally classified pseudo-colored chromosomal images with t(2;2) derivative and other abnormalities was shown in the karyotypes of the metaphases from PC (A), F111 (B), F111-NA16 (C), F111-colony cells (D) and F111-tumour cells (E).

For identification of numerical and structural chromosomal changes we did the spectral karyotype analysis of metaphase chromosomes stained with whole chromosome paints of the rat in all the cell types as described in Methods. The classified karyotypes of all cell types are shown in Figure 2 and the karyotypic formulae of all the cells studied for each cell type are shown in Table 1. Full karyotypes including the inverted DAPI images, G banded images and RGB color assigned, are shown in Additional File 3 Figure S3. PC A16 cells showed a normal karyotype with a 40XY chromosomal complement indicating the veracity of our chromosomal painting methodology. F111 cells showed aneuploidy, which increased remarkably in F111 NA16, colony derived and tumour derived cells. Metaphases analysed from all the cell types showed a t(6;6) robertsonian translocation however, cells with another robertsonian translocation t (2; 2), which were < 15% in F111 A16 cells, increased to approximately 25% in F111 NA16 and upto 100% in colony derived and tumour derived cells. Other chromosomal abnormalities also increased in F111 NA16, colony derived and tumour derived cells. These data clearly depict that cells bearing chromosomal derivative t (2; 2) had a survival advantage in the non-adherent condition. These cells also had the tendency to accumulate other chromosomal abnormalities.

**Table 1:** Chromosomal arrangement in different karyotypes

A16	NA16	Colony Cells	Tumor cells
40, XY, der (6;6),+6 41, XY, der (6;6),+6 41, XY, der (6;6),-20 40,XY, der (6;6),+6,-20 40, XY, der (6;6),-18(1) 40, XY, der (6;6),+6,-11,-18 40, XY, der (2;2),der (6;6),+2 40, XY, der (2;2),der (6;6),+6,-20 40, XY, der (6;6),+6,-18,-20 40, XY, der (6;6),+6,-20(1) 40, XY der (6;6) add (2p), +6, -19,-20 40, XY der (6;6) +7 -12 -20 40,XY, der (6;6), +6,-5,-20 40, XY der (6;6) add (2p), +6, -19, -20 40, XY der (6;6) +7 -12 -20 41, XY der (6;6) add(2p) +6 -5 -20 42, XY, der (2;2),der (6;6), +2,+6	41,XY der (6;6) +2(2),+6,-12,-18,-20(p) Y 41,XY der (6;6) add (2p) +6,-19 41,XY der (6;6) add (2p) +6 40,XY der (2;2) der (6;6) add (2p) -3 40,XY der (6;6) add (2p) +6,-12,-14 40,XY der (6;6) -16 40,XY der (6;6) -20 40,XY der (6;6) add (2p) +6,-11,-16 39,XY der (6;6) add (2p) -3,-9,-11 38,XY der (2;2) der (6;6) +6,-10,-11(2) -20 37,XY der (6;6) +6,-3,-9,-11,-12(2) Y 36,XY der (2;2) der (6;6) +6,-1,-8,-14(2) -20	41,XY der (2;2),der (6;6) +6,+7,-20 42,XY der (2;2),der (6;6) +6,+7,+13,-20 41,XY der (2;2),der (6;6) +6,+7,+19,-3 41,XY der (2;2),der (6;6) +6,+7,-8 41,XY der (2;2),der (6;6) +6,+7,-16 41,XY der (2;2),der (6;6) +6,+7,-18 41,XY der (2;2),der (6;6) +6,+7,+19,-3 40,XY der (2;2),der (6;6) t(9;11) +3, +6,+7,-15,-19,-20 40,XY der (2;2),der (6;6) +6,+12,-1,-16 40,XY der (2;2),der (6;6) +6, -20 39,XY der (2;2),der (6;6) +6,+7,-8,-19,-20 39,XY der (2;2),der (6;6) +6,+7, -8, -19, -20 39,XY der (2;2),der (6;6) t(7;14) +(12;20) +6,+7,-12,-14,-20 39,XY der (2;2),der (6;6) t(1;190),+7,-17, -19 37,XY der (2;2),der (6;6) +6,+7,-9,-13,-14,15,-20 37,XY der (2;2),der (6;6) +6,+7,-18(2), -20	40, XY der (2;2) der (6;6) +2 +6 -18 -20 44, XY der (2;2) der (6;6) t (3;7) +2 +6 +7 -14 43, XY der (2;2) der (6;6) add (5p) +3 +4 +6 +7 +16 -4,20,Y 41, XY der (2;2) der (6;6) (add (4p) del (14p) +2 +6 -15 41, XY der (2;2) der (6;6) +2 +6 +7 -3 -16 41,XY der (2;2),der (6;6) +3,+6,+15, -18, -20 41,XY der (2;2),der (6;6), add (5p) +6,+7, -18 41,XY der (2;2),der (6;6), add (5p),+2,+7, +16, -11, -12, -19, -20 40,XY der (2;2),der (6;6), add(5p), +6,+7, -14, -20 40, XY der (2;2) der (6;6) add (5p) +6 +7 -13 -20 Y 39, XY der (2;2) der (6;6) +2 +6 -16 -18 -19 39, XY der (2;2) der (6;6) t (5;12) +6 +7 -15 -20 Y 38, XY der (2;2) der (6;6) t (6,7) add (7p) +6 -3 -4 -15 -20 38, XY der (2;2) der (6;6) t (7;8) t (12;17) +6 -8 -12 -14 -20, Y

The chromosomal arrangement of different karyotypes from A16, NA16, colony cells and tumour cells.

### RNA profiling and differential gene expression

**Figure 3:**
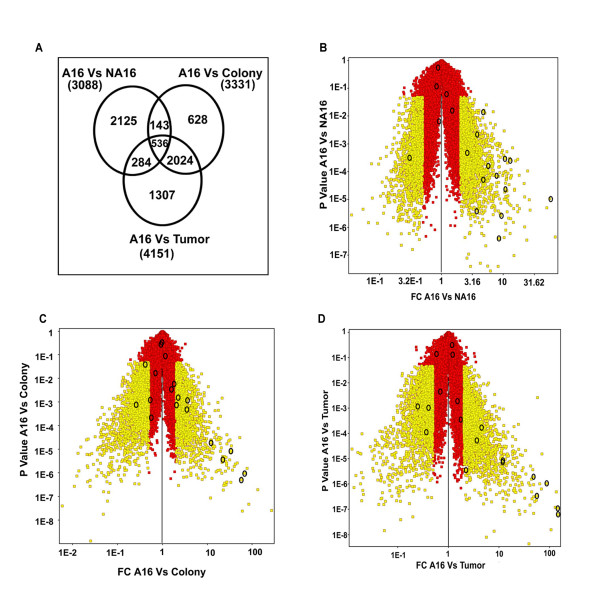
**Venn diagram and Volcano plots representing the summary of microarray results**. Figure A represents venn diagram, where each circle shows the main effects of Avadis analysis: A16 Vs NA16, A16 Vs Colony and A16 Vs Tumour. Numbers inside each compartment represents the number of transcripts that are significant for that effect. The intersections of the sets represent genes with *P *< 0.05 and Fold change 2 for each of the effects involved in the intersection. Note the expansion of transcripts that are significant and high from A16 Vs NA16 to A16 Vs Tumour. B, C and D represent Volcano plot comparison of gene expression between A16 Vs NA16, A16 Vs Colony and A16 Vs Tumour. The *x *axis indicates the differential expression profiles, plotting the fold-induction ratios in a log scale. The *y *axis indicates the statistical significance of the difference in expression (*P *value from a *t *test). Yellow color represents genes with *P *< 0.05 and Fold change 2. Squares within circles show potentially interesting genes from a biological standpoint. The increase in the fold change was observed from A16 to tumor cells and the genes that were obtained within the significant range were analysed through affymetrix database for different process based on functional annotations as shown in the Additional File 5 Figure S4.

RNA was isolated from A16, NA16, colony and tumour-forming F111 cells as described in Additional File 4. F111 A16 RNA was used as reference to obtain the fold change (FC) values in other cell types. Each microarray experiment is repeated biologically and technically twice. Venn diagrams of the number of genes showing up or down regulation with FC value > 2.0 and with p value of < 0.05 have been shown in the Figure 3A. The same results are represented as volcano plots in Figure 3B, C and 3D. As can be seen in the plots, the number of genes affected continues to increase with the process of transformation as shown in Figure 3A. In the up regulated category this increase is represented by the expression of hypoxia related gene Stc1, which increased to a FC value 55.9 in F111 NA16 cells, dropped below the FC value > 2.0 in colony derived cells and again increased to FC value 48.4 in tumor derived cells (Table 2).

**Table 2:** Validation of selected genes by quantitative real time PCR

Pathways	Genes	Microarray			QPCR		
		NA16	Colony	Tumor	NA16	Colony	Tumor
**Hypoxia**	**Hif1a**	NC	4.71	2.08	0.51	1.60	1.78
	**Stc1**	55.90	NC	55.60	873.10	1.01	347.26
	**Vegfa**	-2.5	3.73	NC	10.56	1.37	6.59
	**Vhl**	3.68	NC	NC	6.45	1.12	1.80
	**Ddit3**	8.45	2.14	4.64	11.31	1.52	1.00
	**Egln3**	7.72	12.18	11.58	68.12	22.16	25.46

**Glycolysis/Gluconeogenesis**	**Pfkm**	3.60	-3.8	NC	4.29	1.32	2.35
	**HK2**	4.78	NC	NC	29.02	1.36	3.05
	**Pdk1**	9.11	NC	-2.75	13.00	1.00	2.00
	**Adh1**	10.76	66.9	47.76	4.44	216.77	135.30
	**Aldh3a1**	10.52	3.49	-2.36	3.36	18.38	1.04
	**Slc2a1**	5.80	NC	2.11	27.86	1.64	1.78

**Tumor formation/** **metastasis**	**Spp1**	NC	22.24	145.20	6.19	64.00	139.10
	**Mmp3**	NC	33.52	87.30	0.03	0.04	6.68
	**Egfr**	2.43	-2.46	NC	0.01	0.01	0.10
	**Rb1**	2.48	3.17	3.60	0.69	1.12	2.64

Total RNA was collected from F111-NA16, Colony and tumour cells and validated for the selected genes of Hypoxia, glycolysis/gluconeogenesis and tumour formation/metastatsis from microarray data and were compared with control[F111-A16]. Genes showed the same trend in regulation in the qPCR and microarray experiments, but not necessarily by the same fold change. No change (NC) refers to a gene whose expression was below twofold with *p *Value less than 0.05 for the microarray.

We classified the genes into 14 different pathways using Genmapp and Affymetrix databases (Additional File 5 Figure S4). The information for the functional classification of different pathways is taken from Genmapp and gene annotation list for each pathway was obtained from the Affymetrix. The maximum number of genes alterations (254) were seen in the tumor formation/metastasis category (Additional File 5 Figure S4A large circles) but the maximum quantitative change in gene expression occurred in genes belonging to the hypoxia and glycolysis pathways (Additional File 5 Figure S4A small circles). We validated the expression of some genes in these 3 pathways (depicted as empty circles in Figure 3A-C) in greater detail by using quantitative PCR (Q-PCR). The primers used for this analysis are shown in Additional File 6 Table S1 and the gel pictures of QPCR are shown in Additional File 7 Figure S5. Comparison of the microarray and the QPCR results, as shown in Table 2 clearly validate the microarray data for all the genes that we have analysed.

### Cellular expression of hypoxia related proteins

**Figure 4:**
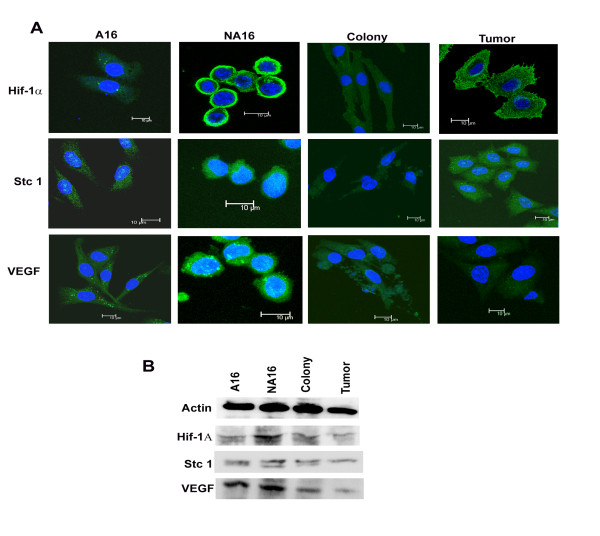
**Expression of Hif-1 α, Stc 1 and VEGF**. The figure includes the immuno-fluoroscence staining of Hif-1 α, Stc 1 and VEGF of A16, NA16, colony and tumour cells respectively (A). Western blot pattern for the expression of actin, Hif-1α, Stc 1 and VEGF, the equal amount of lysates collected from A16, NA16, colony and tumour cells was considered for 10% SDS-PAGE (B). The Figure represents the increased expression pattern with F111 NA16 cells.

Further validation of 3 genes in the hypoxia pathway (Stc1, Hif1α, Vegfa) was done by immunofluorescence staining of cells and immune blotting methods. The results clearly showed the maximum expression of all the 3 proteins in F111 NA16 cells by both methods (Figure 4A&4B) although the colony and the tumor derived cells showed higher protein levels compared to A16 cells. The significance of these results could be reflected in the fact that NA16 cells survive under highly stressful conditions.

### Cancer stem cell like properties

**Figure 5:**
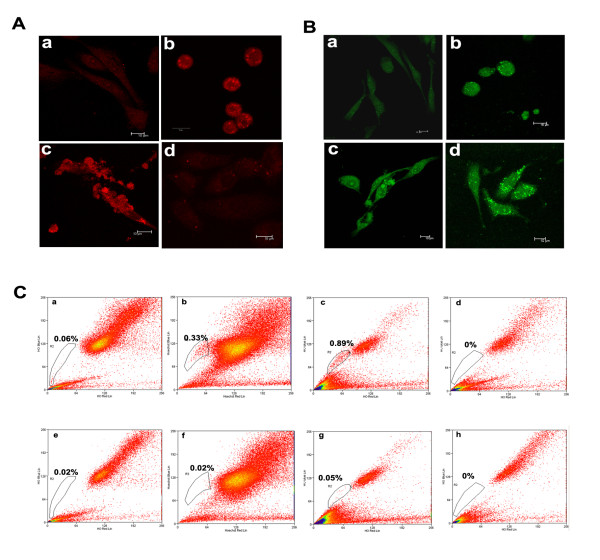
**Cancer Stem cell marker analysis**. Immunofluoroscence of different type of cells derived from F111 cell lines, representing A16 (a), NA16 (b), colony (c) and tumour (d) cells were stained with specific stem cell marker proteins such as ABCG 2 (Figure 5A) and CD133 (Figure 5B). The figure indicates the higher expression for both proteins in F111 NA16 cells. Magnification bars are included in the figure. Figure 5C Flow Cytometry analysis. FACS analysis for side population activity in A16, NA16, colony and tumour cells were done by staining with Hoechst dye (a, b, c, d successively) and their respective inhibition with verapamil treatment (e, f, g, h successively). The Side population community is represented in the gate and was high in F111 NA16 as shown in the figure.

ABCG2 and CD-133 are usually expressed as specific cell surface markers on stem cells. Side population analysis is based upon the stainability of cells with a vital fluorescent dye Hoechst 33342 that enters live cells and quantitatively binds to nuclear DNA. In stem cells the dye is pumped out due to the activity of multi drug resistance (MDR 1-4) family of proteins of which ABCG2 is a member. Due to this stem cells stain poorly with Hoechst 33342 as compared to other cells and this process is inhibited by verapamil which acts on the pump. Our results with confocal microscopy showed an increase in expression of both ABCG2 and CD133 proteins in F111 NA16 cells and in the colony derived cells but expression of both these proteins in tumour derived cells was lower (Figure 5A&5B). Side population (SP) cell analysis of each cell type by flow cytometry also showed an increase in the percentage of SP cells from 0.06% to 0.33% from A16 cells to NA16 and further to 0.89% in colony cells but SP cell number in tumour cells was very low (Figure 5Ca-d). This low percentage of SP in tumour cells could be because the expression of ABCG2 protein was not maintained from NA16 to the tumour stage because of the diversification in there expression pattern. The SP phenotype of these cells was valid because when the cells were treated with verapamil for 30 minutes, prior staining with Hoechst, the SP cell number in each cell type was negligible (Figure 5Ce-h).

## Discussion

Transformation at cellular level refers to the collective changes, such as uncontrolled cell division and morphological alterations etc., which occur to convert a normal cell to cancer cell [15]. In this report we have analyzed the involvement of anchorage independent cell survival in inducing cellular transformation by considering the properties of primary skin fibroblast cultures and the F111 cell line. Upon prevention of adhesion to the substratum these cells entered into apoptosis during which their morphology and gene/protein expression profile were altered significantly. NA cells of both origins showed an incremental increase in the percentage of cells that are committed to die with passage of time that they have spend in the non-adherent state (Additional File 1 Figure S1). In the F111 NA16 cell population, about 20% cells stayed alive and they were rich in apoptotic proteins such as caspase 3 and they also showed DNA fragmentation [13,14]. However, these cells could be rescued as transformed cells if they were plated in soft agar, or injected subcutaneously into nude mice. Thus, F111 NA16 cells which, as shown earlier [13,14], exhibit both cell death and cell survival pathways, represent a transition state between normal and transformed cells, showing a preferential cell survival with a particular karyotype and an altered gene expression profile. Our cells provide a model system to study the relationship between normal cell-matrix adhesion pathways and the steps that lead to the transformation of skin fibroblasts. A somewhat similar model has been reported earlier also where changes in the adhesion state in melanocytes led to their transformation [5] however; there are not many similar reports available in the literature.

### Colony formation in soft agar

Anchorage independent growth of cells is defined further by the ability of cells to proliferate in a semisolid medium [16]. Non adherent cells that can grow in soft agar are considered transformed and those that cannot grow in this condition are considered normal. Thus, colony formation in soft agar is considered to be a transition state of a normal cell to a transformed cell. We have shown here that NA16 cells from both PC and F111 cell types exhibited this transition because both grew as colonies in soft agar however; the colony size for the PC NA16 cells was smaller. Our observation that efficiency of colony formation was highest for both the cell types only after the cells had remained non adherent for 16 h indicated that cells underwent a clonal selection during transformation after experiencing prolonged stress conditions and gene alterations imposed by non-adhesion. The high background of colony formation in F111 A16 cells but not in PC A16 cells indicated that if cells had been maintained in culture for prolonged periods, they would exhibit properties of partially transformed cells without undergoing anoikis, as per the reasoning discussed by Rubin [17]. The inability of F111 A16 (or PC NA16 cells) to make subcutaneous tumours in nude mice is consistent with the earlier reports that murine cells require alterations in at least two collaborative oncogenes such as ras and c-myc (or adenovirus E1A) to exhibit full transformation [18]. We have earlier shown that F111 A16 has less Ras activity than F111 NA16 [13].

### Tumour formation and metastasis in nude mice

When F111 NA16 cells were injected subcutaneously as xenografts into nude mice they grew efficiently as fibrosarcomas, as envisaged by the cell morphology and higher collagen secretions (Additional File 2 Figure S2B-F). The minimum latent period required by these cells to grow as tumours of a measurable size was 4-6 weeks, therefore in this period the injected cells could be considered as '*in vivo *cultures' representing the intermediate step between transformed and tumor forming cells as described by Frese and Tuveson [12]. During this "*in vivo *culture" period the *in vitro *transformed cells acquire additional properties, such as angiogenic activity and tissue invasion capacity, in order to metastasize and re-establish themselves in other tissues. In our system also cells were able to metastasize to distant organs such as lung and liver 8-10 weeks post-injection.

Keeping in view that these metastatic cells were derived by simply rescuing them from the process of anoikis *in vitro *and grafting them subcutaneously in *vivo *we propose that our tumor model system is different from the previously reported models of cancer in which tumors arise either spontaneously or due to various environmental exposures including radiation, chemicals, pathogenic viruses and microbial flora [5,12,19].

### Clonal selection during anoikis induced transformation

One of the important features revealed by our results is that selection of transformed cells within the cell population undergoing anoikis is done on a clonal basis. Comparison of spectral karyotypes from cells at different stages of transformation (Figure 2, Additional File 3 Figure S3 and Table 1)were analysed as described by Buwe et al.,[20]. These results showed that F111 A16 cells were cytogenetically heterogeneous because most cells showed a t (6;6) translocation with or without some other random abnormalities (Table 1); this could be a result of the prolonged maintenance of these cells in culture, however it was not sufficient to make the cell line fully tumorigenic i.e. these cells could make colonies in soft agar but did not make tumors in nude mice. During the process of anoikis and subsequently during growth in soft agar colonies and in xenografted tumors, we observed the selection of another cell type, which contained the t (2; 2) translocation along with the t (6; 6). This abnormality which was seen only in < 15% F111 A16 cells and < 25% F111 NA16 cells was observed in 100% cells obtained from the colonies and tumors. In the latter case it was seen along with a wide range of other random chromosomal abnormalities thereby indicating that cells bearing the t (2; 2) translocation had a survival advantage in the transformed phenotype. The higher range of chromosomal aberrations in the tumor cells indicated that these cells were genetically unstable. Telomere length analysis done by the FISH method showed that telomere length in tumor cells was significantly shorter than in the adherent F111 cells (Additional File 8 Table S2) thus further strengthening this conclusion. It has been reported by others that progressive accumulation of DNA damage leads to the selection of transformed cells [21]. Cell transformation is also described as a cause for increase in the instability and clonal selection in cancers [22-25]. Earlier reports have also indicated that several genes from rat chromosome #2 are involved in the control of tumour growth or development [24] and association of t (2; 7) as a chromosomal marker in the phenotypic transformation of rat cell lines [26]. In summary the genomic instability observed in the transformed cells derived from F111 NA 16 cells is an important step to accumulate mutations that are necessary for cancer development, as is reported for many other cancers [27].

### Hif-1 and its role in regulating gene expression

Gene expression profiles of our experimental cell types indicated that among other pathways, genes related to hypoxia, glycolysis/gluconeogenesis and tumour formation/metastasis pathways are significantly affected in F111 NA16 cells (Table 2). In particular, enzymes from the glycolysis/gluconeogenetic pathway, such as phosphofructokinase (pfkm), hexokinase (Hk2), aldehyde dehydrogenase (Aldh3a), Adh 1, pyruvate kinase (Pdk1) were over expressed in F111 NA16 cells. This could be because F111 NA16 cells in contrast to F111 A16 cells have less oxygen diffusion due to a rapid change in their morphology which leads to a metabolic shift from aerobic to anaerobic glycolysis for ATP generation as has been shown for many cancer cells [28-30]. Another related marker which is up regulated in F111 NA 16 cells was Slc2a1 (Glut 1), a glucose transporter required for glucose uptake for anaerobic glycolysis to sustain the viability (Table 2). It is interesting to note that all these genes are known to be predominantly controlled by the transcription activator protein Hif-1 (hypoxia inducible factor-1) a heterodimeric protein normally found in the nucleus under acute hypoxic conditions by the association of the α and β subunits [31-33]. In our case we could visualize active Hif-1α in the plasma membrane of NA16 cells by immunofluorescence staining. An active Hif-1 further binds to specific hypoxic elements that encodes for VEGF (involved in proliferation and angiogenesis), stanniocalcein (involved in homeostasis of calcium and Pi), carbonic anhydrase IX (regulates cellular pH), Gult-1 and other glycolytic proteins, Rb1(involved in the proliferation during selection of the clonal population), matrix metalloproteases and osteopontin (involved in cancer growth and metastasis), [31-33]. Another target of Hif-1α regulation is the ABCG2 protein, which facilitates the SP phenotype and a stem cell marker, CD133 expression confers the combined effect, for the better survival in hypoxic conditions, were also overexpressed in all the transformed cells [34]. Thus, the gene expression profiles of cells in our model, whether they were NA16, colony derived or tumour derived, support the contention that hypoxia related pathways play role in the selection of transformed cells from within the anoikis resistant cells.

## Conclusion

In summary, we have shown that the process of anoikis and cell transformation can be directly related i.e., if cells are rescued from the cell death pathway induced by anoikis it is possible to obtain transformed cells. At present our observations are restricted to the cell types we have studied, but it should be noted that chemical and physical agents do not easily transform rat fibroblasts and hence rescue from anoikis could act as a natural method for getting fibrosarcomatous tumors in rat fibroblasts.

## Methods

The sources of chemicals and other materials used in the study are described in an Additional File 4.

### Cells and Cell Culture

All experiments were performed using a normal rat (Fischer strain) fibroblast cell line, F111, the origin of which has been described earlier, and primary cultures of skin fibroblast (PC) were made from skin grafts of neonatal rat pups (1-2 days old). F111 cells were maintained in Dulbecco's modified Eagle's medium (DMEM) supplemented with 10% fetal calf serum (FCS), penicillin (100 U/ml) and streptomycin (50 mg/ml) and they were used within five passages after revival from cryo-preservation. PC cells were maintained Dulbecco's modified Eagle's medium (DMEM) supplemented with 20% fetal calf serum, which were used within 2 to 3 passages after plating.

### Preparation of A and NA cells

Adherent (A) and non-adherent (NA) cells from F111 and PC cells were collected at different time points. The method is described briefly in Additional File 4. The type of A and NA cells collected is shown by the suffixed number to these letters which represents the time point (in hours) at which they were collected from the agarose/plastic surface.

### Cell Viability Assay

The cell viability for adherent and non-adherent cells at different time points was measured by using the MTT assay as described earlier [13].

### Soft Agar Colony formation Assay

1×10^5 ^cells in DMEM containing 10% FCS from both F111 and PC cells in both adherent and non-adherent conditions were suspended in 0.3% agarose. Then, this mixture was layered onto a solidified 1%agarose layer and incubated at 37°C, 5% CO_2_. The assay was repeated 5-6 times, and colonies each containing > 50 cells, were counted after a week of incubation.

### Tumour formation in nude mice

5×10^6 ^A16 and NA16 cells from PC and F111 cells were collected at different time points in PBS were injected subcutaneously in to a 4-6 week old, homozygous nude mice (NIH strain, Nu/Nu). The size of the tumor was measured at weekly intervals. All experimental procedures were approved by the Institutional Animal Ethical Committee (IAEC 132/2007 dated May 19^th ^2008).

### Isolation of colony cells and tumour cells

The colonies embedded in soft agar were collected in DMEM containing 10% FCS by disrupting the agar; the cells were allowed to adhere on a 70 mm petri dish for a short period (approximately 4 hr). The attached cells were processed for further experiments. Cells from tumors were collected by treatment of small tumor explants with 0.1% trypsin-EDTA for 30-40 minutes and layered in 20% serum containing DMEM medium with 5% CO_2_. Cells from the explants were released after a week was used as source of tumour cells.

### Histopathology

Excised tumours and the corresponding lung and liver tissues were collected from nude mice and fixed in 10% formalin. The tissues were dehydrated gradually in alcohol; embedded in paraffin and were sectioned at a thickness of 5 μm. The tissue sections were stained with Haematoxylin and Eosin and Masson-Trichome for collagen. The details of the methods were described in Additional File 4.

### Karyotyping of chromosomes by using Rat SKY probes

Metaphases from PC cells, normal F111, NA16, Colony and tumour cells were dropped on a clean glass slide (Fisher scientific, USA) was which was used for chromosomal painting. The detailed method is described in Additional File 4.

### Side Population (SP) cell analysis

One million single cells from all cell types were incubated with 2.5 μg/mL Hoechst 33342 (Sigma) for 90 minutes at 37°C. Cells were collected by centrifugation at 2000× g and washed once in HBSS (Hank's balanced salt solution) and re-suspended in ice-cold HBSS, Propidium iodide (50 μg/ml) was added just before analysing the cells on a MoFlo cell sorter (Dako Cytomation Glostrup, Denmark). Inhibitory action on the Hoechst efflux was checked with 50 mM Verapamil.

### Immunoflurescence and immunoblotting

Single cell suspensions, of all cell types were plated on coverslips and fixed in 70% ethanol followed by three washes with PBS. Mild cell permeabilization was done with 0.1% triton-X 100 and staining for Hif 1α (1:100), VEGFa (1:150), Stc1 (1:200), ABCG2 (1:50) and CD-133 (1:100) was done by incubating cells at 4°C for 1 hr with the corresponding primary antibodies. After washing, the cells were treated with appropriate fluorescent-labelled secondary Ig antibodies. Stained cells were observed under a Leica confocal microscope with a 63 X, Plan Apo objective. Immunoblotting was performed by analysing equal amount of protein lysates (prepared in lysis buffer containing 50 mM Tris buffer, pH 7.5, 150 mM NaCl, 1 mM EDTA, 1 Mm EGTA, 1 mM Na_3_VO_4_.H_2_O) on a 10% SDS-PAGE proteins were blotted on to nitrocellulose paper and probed with different antibodies such as Hif-1α, VEGFa, Stc1 which were visualized by enhanced chemi-luminescence.

### RNA extraction and Microarray Analysis for differential gene expression

Total RNA collected from F111-A16, NA16, Colony and Tumour cells was used to screen for differential gene expression patterns The method for the extraction of RNA and microarray analysis has been provided in Additional File 4. The files for the microarray analysis of all the four cell types has been deposited in the Gene Expression Omnibus (GEO) database and can be publically accessed via the accession number GSE24893.

## Abbreviations

PC: Primary culture of skin fibroblasts; A16: Adherent cells at 16 h time point; NA16: Non-adherent cells at 16 h time point; Hif1a: Hypoxia inducible factor 1 alpha; Vegfa: Vascular endothelial growth factor alpha; Stc1: Stanniocalcin1; H&E: Haematoxylin and Eosin.

## Competing interests

The authors declare that they have no competing interests.

## Authors' contributions

JR designed the manuscript and wrote the earlier drafts, carried out the tissue culture, flow cytometry, tumor and western blot experiments and scrutinized the results of all other experiments; RK assisted in tissue culture, soft agar assay, tumor assays, and did western blots, immunofluorescence and flow cytometry experiments and also helped in writing the final manuscript; SP did the microarray & RT PCR experiments; ART assisted in the histology and tumor experiments; JMK did the animal maintenance and monitored tumor growth and histology; LR did all the chromosomal analysis; GP wrote the final manuscript, scrutinized the experimental results and arranged the funding for the paper.

All authors read and approved the final manuscript.

## Author Information

Nil

## Supplementary Material

Additional file 1 **Figure S1: Cell and colony morphologies**. The figure shows cell morphologies of F111 A16 and PC A16 cells during adherent **(a & b) **and rounded F111 NA16 and PC NA16 cells during non-adherent conditions **(c & d)**. Panel's **e & f **show the morphologies of colonies derived by plating F111 NA16 and PC NA16 cells in soft agar after 7 days. The insert showed the higher magnification of the colony with more number of cells and increased size of the colony with F111 NA16 cells. Click here for file

Additional file 2 **Figure S2: H&E and Masson-Trichome staining of 10-12 week old tumors and other tissues**. H&E staining of tumors and the corresponding lung and liver tissues is shown in panels **A-C **respectively. Masson-Trichome staining for collagen for the same sections is shown in panels **D-F **respectively. Transformed cells can be identified as pleomorphic, spindle shaped cells invading into muscular region **(A)**. The crescent shaped nucleus in the spindle shaped neoplastic cells is indicated with arrows in the inset of panel A (magnification 100×); cytoplasm is stained dark red and it is surrounded by blue colored connective tissue (collagen) as shown in panel D. Metastatic tumor cells present in lung (B&E) and liver tissues (C&F) have been indicated with an arrow in the insets of the corresponding panels. Click here for file

Additional file 3 **Figure S3: Detailed karyotypes of all the cell types**. Pictures of metaphases showing inverted images of DAPI stained chromosomes (left), RGB colored (centre), and spectrally classified pseudo-colored chromosomes (right) are shown for all the cells types studied. The panel descriptions are: PC cells **(A) **F111 A16 cells **(B) **F111 NA16 cells **(C) **colony derived cells **(D) **and tumor derived cells **(E)**. Various chromosomal abnormalities and t(2;2) chromosomal derivative was observed in F111 NA16, F111 colony and F111 tumour cells. Click here for file

Additional file 4 **Materials and detailed Methodology**. Click here for file

Additional file 5 **Figure S4: Classification of differentially expressed genes into pathways**. Using the Affymetrix database, the number of genes showing a change in their expression level (indicated on the Y axis of each bar chart) in F111 NA16, colony derived and tumour derived cells was calculated in relative to the F111 A16 cells. The genes were classified into 14 categories as indicated on the X-axis. **Panel A **represents the total number of genes analysed, with high number of genes which were associated for tumor formation and metastasis, **Panel B **represents the number of up regulated genes, which were increased in F111 NA16 cells and **Panel C **represents number of down regulated genes. The encircled gene categories were analysed further by QPCR and immunofluorescence validation. Click here for file

Additional file 6 **Table S1: Primers used for RT-PCR analysis**. Click here for file

Additional file 7 **Figure S5: Q-PCR analysis of selected genes**. The figure shows gel profiles of the amplified RNA of the indicated genes in the hypoxia, glycolysis/gluconeogenesis and tumour formation/metastasis pathways in the cell types as indicated. The figure shows the relatively high expression of the selected genes only in F111 NA16 cells. β-actin RNA was used the loading control. Click here for file

Additional file 8 **Table S2: The Length of Telomere for all the cell types**. Click here for file
