# Supplementary figures and images for: Differential gene expression and clonal selection during cellular transformation induced by adhesion deprivation
Source: BMC Cell Biol. 2010 Dec 2;11:93. doi: 10.1186/1471-2121-11-93 (PMC3012028; doi:10.1186/1471-2121-11-93)

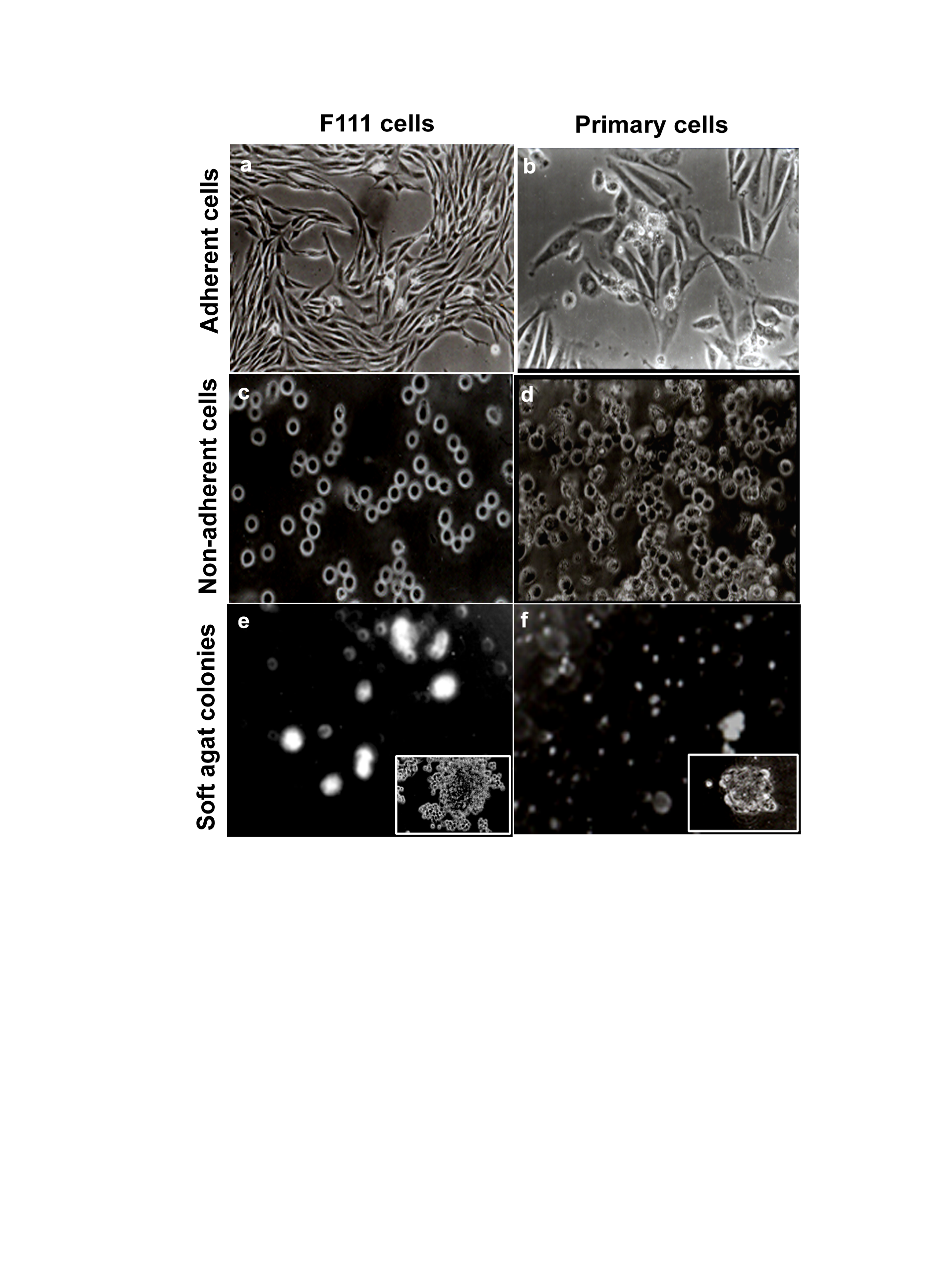

Supplement: Additional file 1 — Figure S1: Cell and colony morphologies. The figure shows cell morphologies of F111 A16 and PC A16 cells during adherent (a & b) and rounded F111 NA16 and PC NA16 cells during non-adherent conditions (c & d). Panel's e & f show the morphologies of colonies derived by plating F111 NA16 and PC NA16 cells in soft agar after 7 days. The insert showed the higher magnification of the colony with more number of cells and increased size of the colony with F111 NA16 cells. [file 1471-2121-11-93-S1.TIFF]

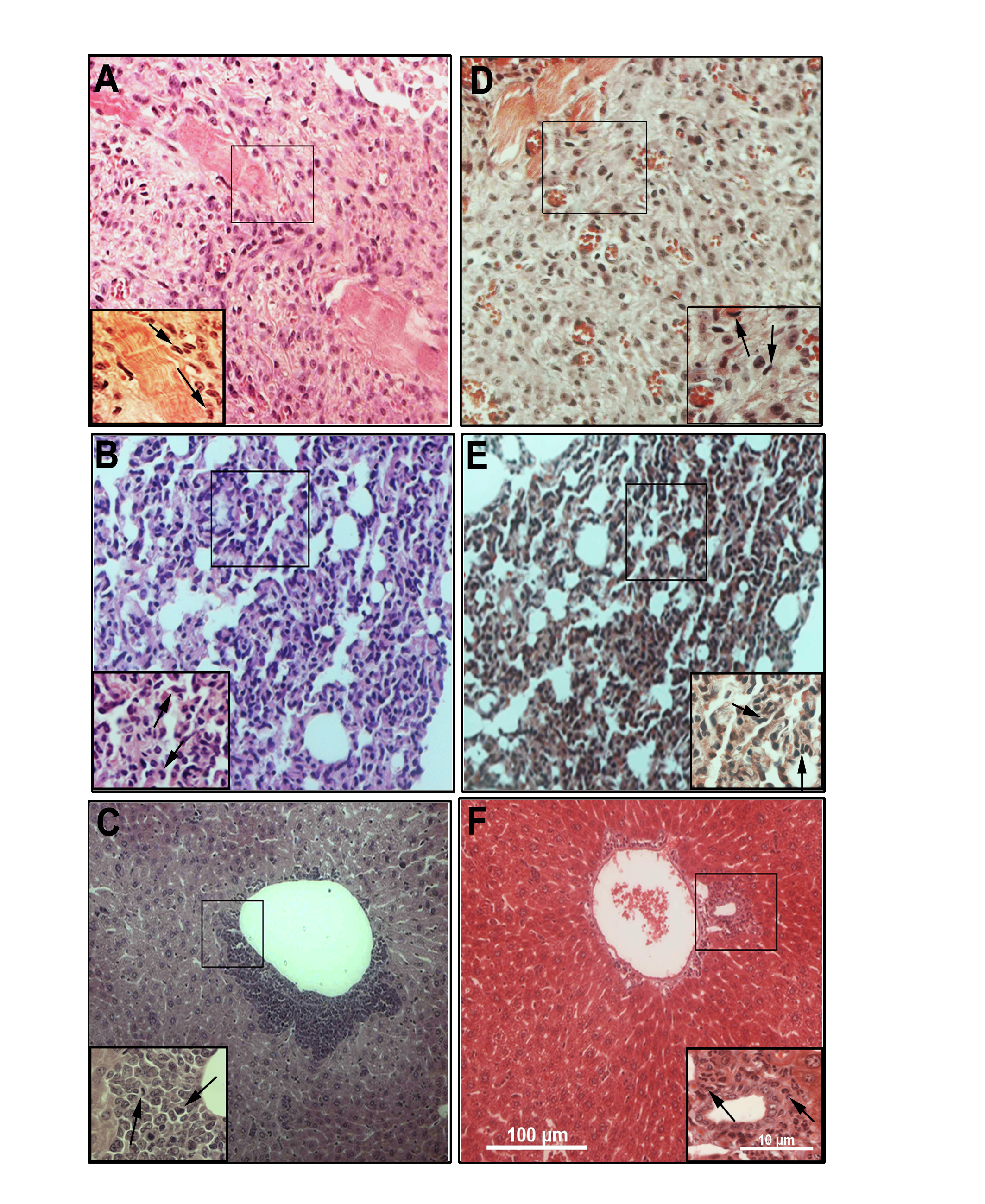

Supplement: Additional file 2 — Figure S2: H&E and Masson-Trichome staining of 10-12 week old tumors and other tissues. H&E staining of tumors and the corresponding lung and liver tissues is shown in panels A-C respectively. Masson-Trichome staining for collagen for the same sections is shown in panels D-F respectively. Transformed cells can be identified as pleomorphic, spindle shaped cells invading into muscular region (A). The crescent shaped nucleus in the spindle shaped neoplastic cells is indicated with arrows in the inset of panel A (magnification 100×); cytoplasm is stained dark red and it is surrounded by blue colored connective tissue (collagen) as shown in panel D. Metastatic tumor cells present in lung (B&E) and liver tissues (C&F) have been indicated with an arrow in the insets of the corresponding panels. [file 1471-2121-11-93-S2.TIFF]

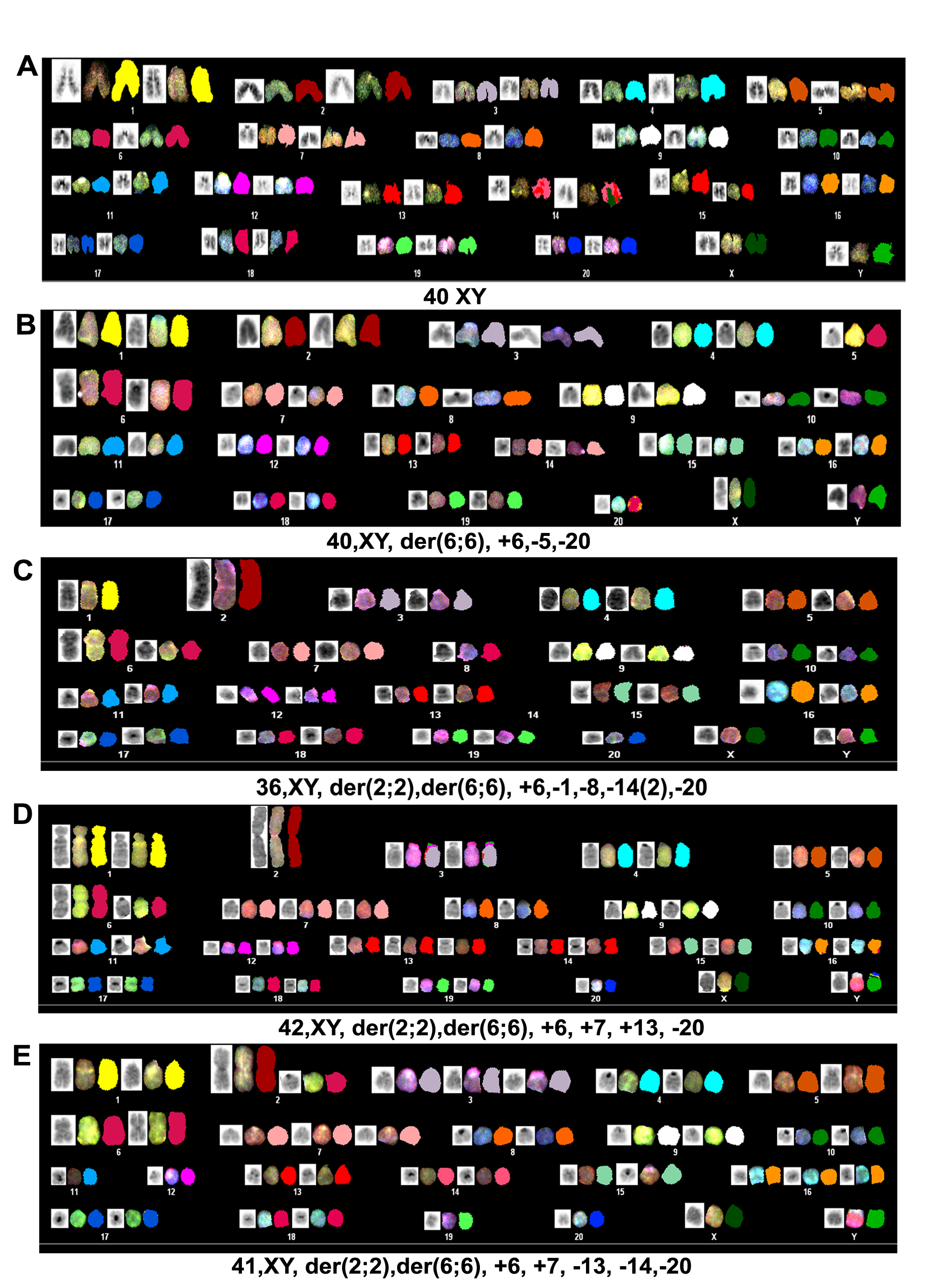

Supplement: Additional file 3 — Figure S3: Detailed karyotypes of all the cell types. Pictures of metaphases showing inverted images of DAPI stained chromosomes (left), RGB colored (centre), and spectrally classified pseudo-colored chromosomes (right) are shown for all the cells types studied. The panel descriptions are: PC cells (A) F111 A16 cells (B) F111 NA16 cells (C) colony derived cells (D) and tumor derived cells (E). Various chromosomal abnormalities and t(2;2) chromosomal derivative was observed in F111 NA16, F111 colony and F111 tumour cells. [file 1471-2121-11-93-S3.TIFF]

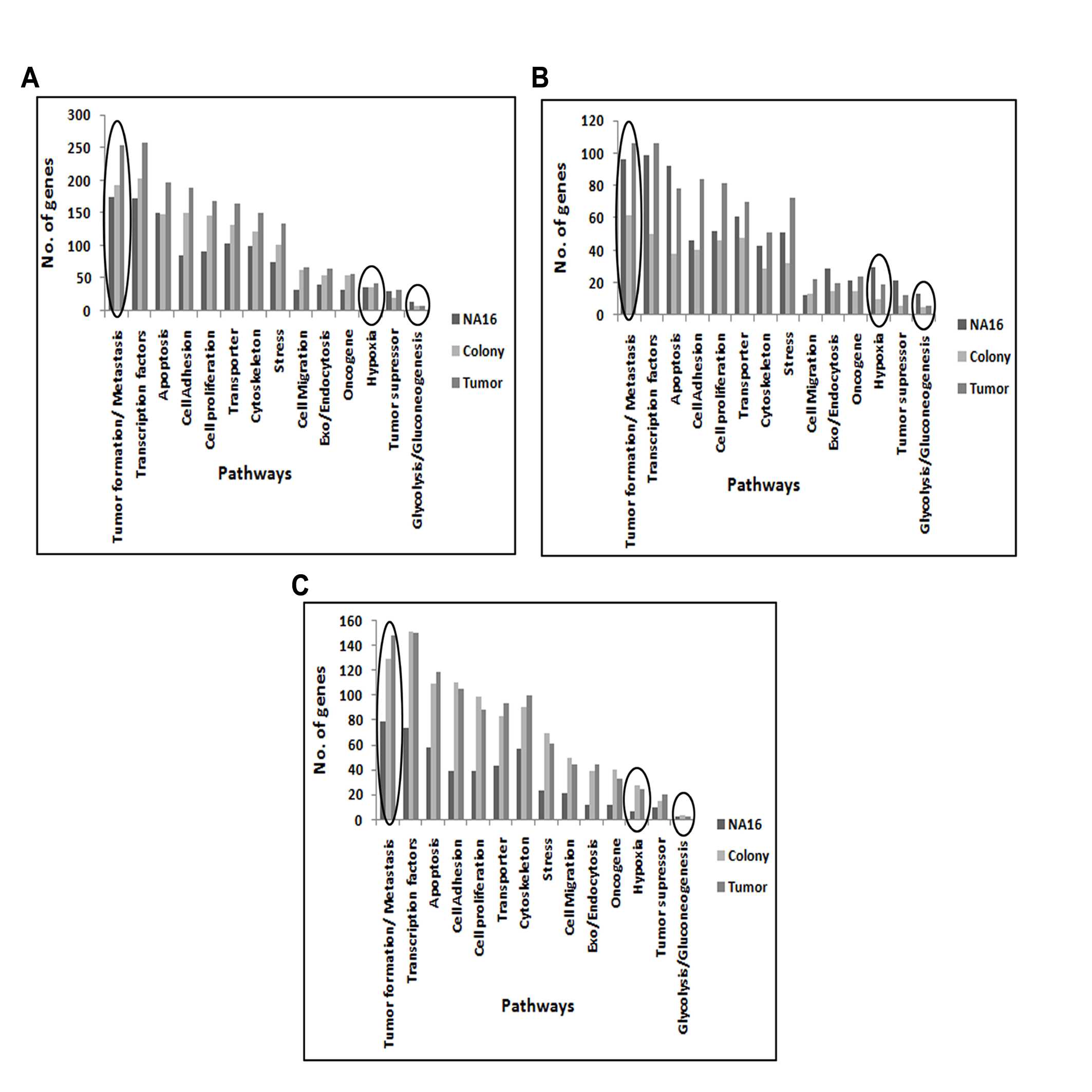

Supplement: Additional file 5 — Figure S4: Classification of differentially expressed genes into pathways. Using the Affymetrix database, the number of genes showing a change in their expression level (indicated on the Y axis of each bar chart) in F111 NA16, colony derived and tumour derived cells was calculated in relative to the F111 A16 cells. The genes were classified into 14 categories as indicated on the X-axis. Panel A represents the total number of genes analysed, with high number of genes which were associated for tumor formation and metastasis, Panel B represents the number of up regulated genes, which were increased in F111 NA16 cells and Panel C represents number of down regulated genes. The encircled gene categories were analysed further by QPCR and immunofluorescence validation. [file 1471-2121-11-93-S5.TIFF]

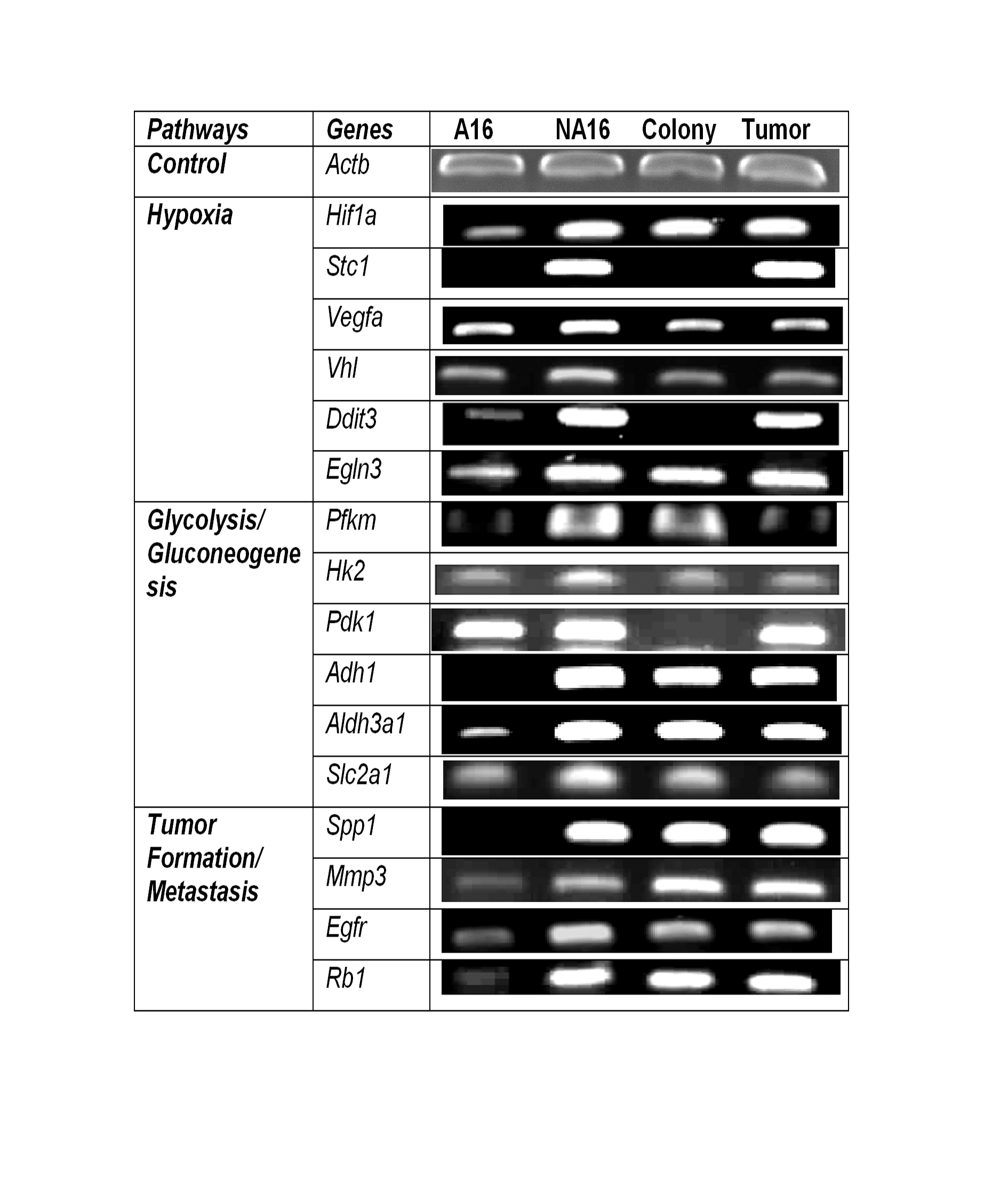

Supplement: Additional file 7 — Figure S5: Q-PCR analysis of selected genes. The figure shows gel profiles of the amplified RNA of the indicated genes in the hypoxia, glycolysis/gluconeogenesis and tumour formation/metastasis pathways in the cell types as indicated. The figure shows the relatively high expression of the selected genes only in F111 NA16 cells. β-actin RNA was used the loading control. [file 1471-2121-11-93-S7.TIFF]
