# Supplementary material for: Differential gene expression and clonal selection during cellular transformation induced by adhesion deprivation
Source: BMC Cell Biol. 2010 Dec 2;11:93. doi: 10.1186/1471-2121-11-93 (PMC3012028; doi:10.1186/1471-2121-11-93)
Supplement: Additional file 4 — Materials and detailed Methodology. [file 1471-2121-11-93-S4.DOC]

**Additional File 4: Materials and detailed Methodology.**

**SM1.Chemicals.** Kits for Spectral karyotype were purchased from Applied Spectral Imaging, Migdel Ha’Emek, Israel, mini RNeasy spin column RNA purification kit was purchased from Qiagen, USA), Rat 230_ 2 gene chips were purchased from Affymetrix, Inc. (U.S), monoclonal antibody for Vegfa is from Abcam, Cambridge, UK; Stc1 from Santa Cruz Biotechnology; Hif 1α (Clone ESEE 122) is from Life Span Biosciences. Western blot chemiluminescence detection kit was from Boehringer Mannheim. All buffers were prepared from analytical grade reagents available from standard companies.

**SM2.**Both F111 and Primary cells were trypisinzed and suspended in DMEM containing 10% FCS, and plated on agarose (for Non-adherent cells) and uncoated plastic (for Adherent cells) at a cell density of 2-3x10^6^ cells/5 ml and incubated at 37°C and 5% CO2.Adherent cells on the plastic surface were denoted as ‘A’ cells and those from the agarose surface as “NA” cells. The time after the initial plating (in hours), after which the cells were collected, is denoted as a suffix to the letters “A” or “NA”.

**SM3.**Tumors along with other tissues were collected after 10-12 weeks post injection. The tissues were fixed in 10% formalin and then dehydrated gradually in alcohol. The tissues were embedded in paraffin and sectioned at a thickness of 5µ. The H &E and Mason trichome staining was done, the brief protocol is described below, and the section was examined under the microscope.

**H&E staining for nuclear organization in tumor sections.** The thin sections collected on glass slides were deparaffinised with xylene and hydrated with water and then stained with Mayer’s hematoxylin for 15 minutes. Subsequently washed in running tap water for 20 minutes and then counterstained with eosin for 15 seconds. The excessive eosin was removed by dehydrating in 95% and 100% alcohols for two times and in xylene for another two changes for 2 minutes each. The slides were mounted in permount and observed under the microscopy.

**Mason Trichome Staining. S**taining for collagen was done using deparaffinised sections stained with Bouin’s solutions (containing saturated Picric acid, Bie brisch scarlet, acid fuschin and glacial acetic acid) for 1 hour at 56°C. After washing thoroughly, slides were stained with Weigert’s hematoxylin (1% hematoxylin in 95% alcohol) for 10 minutes, washed thoroughly with water and stained further with Biebrisch scarlet acid –fuchsin for 2 minutes. The acidified sections were washed with water and placed in phosphomolbdic-phosphotungstic acid solutions (2.5% of phosphomolbdic acid and 2.5% phosphotungstic acid) for 15minutes and then stained with aniline blue (25g in 20ml of acetic acid) for 5 minutes and fixed in 1% acetic acid. The sections were dehydrated with 95% and 100% alcohol ,xylene and mounted with synthetic resin.

**SM4.Spectral karyotyping**

**Preparation of rat metaphase chromosomes.** Metaphase spreads from adherent (A16), non-adherent (NA16), colonies and tumor derived cells were prepared by treating with 0.03% colchicine according to standard techniques. After hypotonic treatment with -.075% M KCl for about 30 min, the cells were fixed in chilled methanol: glacial acetic acid (3:1). The suspension was dropped onto cleaned wet slides and air-dried. Prior to hybridization, slides were pretreated with 1% formaldehyde and then dehydrated through an alcohol series. For all hybridization freshly prepared slides were used.

***In situ* hybridization and detection.** Rat SKY probe denaturation was done for 7 min at 80°C, followed by pre-annealing for 1 h at 37°C.Slides with metaphase spreads were denatured in 70% formamide, 2X SSC for 90 s at 70°C. For hybridization 3.5µl probe cocktail was applied to denatured metaphase preparations and under a 18x18 mm Coverslip. After hybridization at 37°C for 2 days, slides were washed and hybridization with probe sequences was detected following the manufacturer’s protocol. Chromosomes were counterstained with DAPI and embedded in an antifade reagent (para phenlene-di-amine) and examined under the microscope.

**Spectral imaging.** Fluorescent probe signals were visualized using a 63×-oil immersion objective on a Zeiss Axiophot microscope equipped for epi-fluorescence. For dye excitation the light of a 75 W Xenon lamp was directed through a custom designed SKY filter set (Chroma Technology, Brattleboro, USA). Spectral images were acquired using an SD200-H spectral bioimaging system (Applied Spectral Imaging, Israel) and analyzed by the respective software package Sky View Version.1.6.

**Spectral karyotyping.** For SKY analysis, slides were hybridized using the SKY method according to the manufacturer's protocol (Applied Spectral Imaging, Migdal Ha'Emek, Israel). Images were acquired with an SD300 Spectra Cube (Applied Spectral Imaging) mounted on a Zeiss Axioplan microscope using a custom-designed optical filter (SKY-1; Chroma Technology, Brattleboro, VT). Approximately 20 metaphases were analyzed for each sample. SKY is somewhat limited in the determination of breakpoints and in the identification of intrachromosomal changes such as duplications, deletions, and inversions. The changes on the SKY-painted chromosomes were determined by comparison of the corresponding DAPI staining and inverted image of the corresponding cell.

**SM5.Differential gene expression profile by using microarray**

**RNA extraction and Microarray Analysis.** Total RNA were isolated from A16, NA16, colony derived and tumor derived cells using the mini RNeasy spin column RNA purification kit (Qiagen, Hilden). The yield of RNA was determined by measuring the absorbance at 260 and 280 nm using spectrophotometer (Nanodrop-1000, USA). 15 µg of RNA for each sample was converted to biotinylated cRNA and hybridized to the Affymetrix (Santa Clara, USA) Rat Gene Chip 230_2 array according to manufacturer's protocol. Rat 230_2.0 Gene chips contain 31,000 probe sets represented by 11 pairs of 25mer oligonucleotides. Each probe pair consists of a perfect match oligo (PM) complementary to the cRNA target sequence, and a mismatch oligo (MM) with a single base change in the middle to control for background and nonspecific hybridization. The scanned images were analyzed using Gene Chip Operating Software 2.1 (Affymetrix, USA) to make standard adjustments for artifacts, noise, and background and to calculate a detection *P* value for each gene on each array. The analysis was performed biologically and technically twice with similar results. Normalization of raw data was done using Avadis Software through Plier and RMA algorithms.

**Gene expression analysis by RT-PCR.** Single-stranded cDNA was synthesized using oligo- (dT) primer and AMV reverse transcriptase (Promega, USA). PCR reactions were done in 25 μL volumes and amplified for 2 minutes at 95°C for initial denaturation, followed by 30 cycles at 94°C for 30 seconds, 58°C for 30 seconds, and 72°C for 1 minute (the conditions of reaction cycles and annealing temperatures were optimized for each individual pair of primers). PCR products were separated on 1.0 – 1.5% agarose gels and visualized by ethidium bromide staining. Gene-specific primers for PCR products were designed by Amplifx software (Nicolas Jullien, France) using information from GenBank (NCBI). β-actin was used as an internal control to confirm equal amounts of template.
