# Supplementary material for: Differential gene expression and clonal selection during cellular transformation induced by adhesion deprivation
Source: BMC Cell Biol. 2010 Dec 2;11:93. doi: 10.1186/1471-2121-11-93 (PMC3012028; doi:10.1186/1471-2121-11-93)
Supplement: Additional file 6 — Table S1: Primers used for RT-PCR analysis. [file 1471-2121-11-93-S6.DOC]

**Table S1: Primers used for RT-PCR analysis.**

| ***Genes*** | **Forward Primer (5' - 3')** | **Length** | **Reverse Primer (5' - 3')** | **Length** |
| --- | --- | --- | --- | --- |
| ***Actb*** | GGCCAACCGTGAAAAGATGA | 20 | TCACAATGCCAGTGGTACGA | 20 |
| ***Vegfa*** | TGGACCCTGGCTTTACTGCT | 20 | CGCTGGTAGACGTCCATGAA | 20 |
| ***Vhl*** | GAACTGTTTGTGCCATCCCT | 20 | CTCCGTACAACCTGAAGGCA | 20 |
| ***Egln3*** | TATGTCAAGGAGCGGTCCAA | 20 | GATACAGCGGCCATCACCATT | 21 |
| ***Ddit3*** | CCTGTCCTCAGATGAAATTGGG | 22 | AGCTAGGGATGCAGGGTCAA | 20 |
| ***Hif1a*** | CATCAAGTCAGCAACGTGGAAG | 22 | AAGCACGTCATAGGCGGTTTCT | 22 |
| ***Stc1*** | TGACACAGATGGGATGTACGA | 21 | GGCGATGCACTTTAAGCTCT | 20 |
| ***Pfkm*** | ACTGAGCTGAAGGACCAGACA | 21 | TCTCGTACTTGGCCAGGATT | 20 |
| ***Hk2*** | TTTGGTCTCGTGGACTAAGGG | 21 | ACCACGGCCACAATGTCAAT | 20 |
| ***Pdk1*** | TGAGAATGCGAGACGGCTTT | 20 | CCACTTGTATTGGCTGTCCT | 20 |
| ***Adh1*** | ACACCAAACCCATCCAGGAAGT | 22 | ATGGCAGCTTAACAGGGCAGAA | 22 |
| ***Aldh3a1*** | TGGTCCTTGTCATAGGTGCTTG | 22 | TTCTGAGGGCTTGAGGATCACT | 22 |
| ***Slc2a1*** | ATCGTCAACACGGCCTTCACT | 21 | ATGGTCATGAGCACAGCACA | 20 |
| ***Spp1*** | GTGGTTTGCTTTTGCCTGTTCG | 22 | GTGGCTACAGCATCTGAGTGTT | 22 |
| ***Mmp3*** | AGGCATTGGCACAAAGGTGGAT | 22 | TGTGGGTCACTTTCCCTGCATT | 22 |
| ***Rb1*** | AAAGGTGCCCGATCATGTCAGA | 22 | AAGATGCAGATGCCCCAGAGTT | 22 |
