# Supplementary material for: Differential gene expression and clonal selection during cellular transformation induced by adhesion deprivation
Source: BMC Cell Biol. 2010 Dec 2;11:93. doi: 10.1186/1471-2121-11-93 (PMC3012028; doi:10.1186/1471-2121-11-93)
Supplement: Additional file 8 — Table S2: The Length of Telomere for all the cell types. [file 1471-2121-11-93-S8.DOC]

**Table S2: The Length of Telomere for all the cell types.**

| **Cells** | **TELOMERE LENGTH** |
| --- | --- |
| A16 | **0.96 ± 0.30** |
| NA16 | **1.23 ± 0.64** |
| COLONY | **1.39 ± 0.51** |
| TUMOUR | **0.79 ± 0.29** |
